# Experimenting With Algorithms and Memory-Making: Lived Experience and Future-Oriented Ethics in Critical Data Science

**DOI:** 10.3389/fdata.2019.00035

**Published:** 2019-10-01

**Authors:** Annette N. Markham, Gabriel Pereira

**Affiliations:** Department of Digital Design and Information Studies, Aarhus University, Aarhus, Denmark

**Keywords:** algorithms, critical data science, arts based research, memory-making, ethics, lived experience

## Abstract

In this paper, we focus on one specific participatory installation developed for an exhibition in Aarhus (Denmark) by the Museum of Random Memory, a series of arts-based, public-facing workshops and interventions. The multichannel video installation experimented with how one memory (Trine's) can be represented in three very different ways, through algorithmic processes. We describe how this experiment troubles the everyday (mistaken) assumptions that digital archiving naturally includes the necessary codecs for future decoding of digital artifacts. We discuss what's at stake in critical (theory) discussions of data practices. Through this case, we offer an argument that from an ethical as well as epistemological perspective critical data studies can't be separated from an understanding of data as lived experience.

## Introduction

In recent years, Google Photos and Apple Memories made headlines by promising to cut through the clutter of people's big data by automatically curating our most meaningful photos and videos. These services rely on machine learning and algorithmic processing of data. Far from neutral, these algorithmic services play a key role in how people enact and make sense of their everyday lives. Whether we use Helen Kennedy's phrase to describe this phenomenon as a form of “new data relations” (Kennedy, 2016) or Cheney-Lippold's “algorithmic identities” (Cheney-Lippold, 2011), algorithms are woven into everyday life at the most intimate levels (Gregg, 2011). As Markham (2015) puts it, this intimacy is one that we can see through the lens of a personal relationship, since algorithmic systems function as interpersonal “participants in a continual symbolic interaction process whereby our understandings of self, other, and our social worlds are co-constituted” (p. 5).

We agree with other critical data studies scholars (e.g., Kitchin and Lauriault, 2014; Iliadis and Russo, 2016) that laying out the granularity of how data is generated or represented is important because data analytic processes wield significant and often hidden power in shaping future knowledge, historical legacies, and social formations. As citizens go about their everyday lives and also reflect on various aspects of their lived experience, the power of data analytics presents a “seductive allure” of being “speedy, accessible, revealing, panoramic, prophetic, and smart” (Beer, 2019). As participatory action researchers, we are bridging the academic and public spheres to facilitate general users' knowledge around the idea, not uncommon among critical data scholars, that these “assemblages of data” are co-creators of future imaginaries, acting with moral agency to, as Martin (2018) notes, “silently structure our lives” (p. 2). Within this ecology, as Markham et al. (2018) emphasize, “The locus of responsibility and accountability for ethical design, behavior, and outcomes is difficult to ascertain” (p. 1). We use the example of an artistic video installation we built called *Memory Glitch* to highlight this difficulty. Through three algorithmic transformations of an elderly woman's interview about her experiences in the second World War, we consider how future memories are impacted by algorithmic rewriting of the codecs, or formulas for encoding and decoding data formats. When and where this happens will of course vary: imagining the long future, it could be caused by data loss as physical memory storage devices decay; in the more immediate future, it could be within the automated memory management processes of organizing, prioritizing, and otherwise “curating” a file. It is by now a familiar criticism of algorithmic processes that multiple stakeholders and agents, human, and nonhuman, operate in these systems.

To this ongoing conversation we add the suggestion that focusing critical ethical attention on the algorithmic management of memory and meaning in unexpected ways can enhance the practices of critical data science. We do this partly by foregrounding the fragility of a person's recorded lived experience as it is algorithmically filtered, morphed, transformed, or otherwise remixed. But we seek to go beyond current scholarly refrains that digital archives are precarious, data modeling is flawed, or algorithms are biased. Instead, we build a case for using arts-based and personalized interventions as a way of enabling end users to better “apprehend (theorize, imagine),” in the words of Magalhães (2018, p. 3), the implications and moral agency of algorithmic processes in their everyday lives.

We have been studying these issues through the *Museum of Random Memory* (MoRM), a series of arts-based, public-facing experiments. Over 3 years we have conducted eight workshop/exhibitions in five countries to help people investigate how automated data-related processes might be influencing their own personal and cultural memories. This becomes a study of complex entanglements of lived experience, digitalization of memories, and algorithmic logics. MoRM is an interventionist action, involving an international group of artists, data scientists, filmmakers, computer scientists, scholars, activists, museum curators, lawyers, and university administrators. The eight experiments performed by MoRM have taken different paths of inquiry: some have focused on showing citizens how their digital traces are tracked as they search for things using a browser; others focus on complicating where and how memory is located in everyday analog/digital/data objects.^1^

A large part of the MoRM goal is to combine critical (theory) data studies with a future making orientation and to add examples that illustrate the importance of an ethic of care^2^ in data science practices. The larger project critiques and imagines alternatives to normative ways of working and thinking through data. We believe that there is a troubled and important set of relationships to explore between humans, their data, digital platforms, machine learning trends, and multiple external stakeholders with political and economic interests. What scholar-activist roles can we take to intervene in these often taken-for-granted datascapes?

In what follows, we focus on *Memory Glitch*, a specific MoRM installation developed for exhibition at the *Affects, Interfaces, Events* conference, August 28–30, 2018 in Aarhus, Denmark. The multi-channel video installation experimented with how the memory of one person, Trine, can be decoded and rendered in three very different ways, through algorithmic processes. We describe how this experiment highlights visually and evocatively the everyday (mistaken) assumptions that digital archiving naturally includes the necessary formulas for future decoding of digital artifacts. We conclude by discussing what's at stake in critical (theory) discussions of data preservation practices.

## Meeting the “Data”

It started as a conversation. One morning, as Trine was returning a book to the library, she walked by our MoRM exhibit and heard the MoRM researchers ask passersby to “donate a memory, a random memory, something you want to remember or forget.” She went home, collected her artifact, and returned later that afternoon to donate her memory. The physical artifact she brought was a photocopy of some newspaper clippings where she, alongside some others, was featured as a jazz singer. The memory she wanted to donate, however, was quite different:

*I want to donate the memory of the Germans occupying my home town in Northern Jutland when I was a little girl*.

As with other participants, we invited Trine to spend some time with a MoRM researcher to talk about her memory. Sitting with her in a cozy space, one of us asked Trine why she felt this memory was important, as well as how she thought digital preservation might influence what future archeologists might find if they dug up artifacts from 2017. As the conversation was being filmed, the researcher wrote a few sketchy notes on what Trine was saying:

*Growing up during German occupation in Northern Denmark. ‘People helped each other'. ‘And we're losing that'. ‘Poor, rich, didn't matter'. ‘We oldies talk a lot about it when we meet at the bus stop'. It's boring to ride the bus (esp. 4-6 pm), and ‘they never get up for you—even if you have a limp'*.

Trine reminded us repeatedly that it was crucial to make people remember this time period of Danish history. She expressed concern that “digital media make it more difficult for people to have conversations about the old days,” and how “nobody really talks to each other anymore because they're busy on their phones.”

Like many other participants at this exhibition, Trine spent far longer than we anticipated: 3 h. With Trine's approval, we made an audiovisual recording of her conversation. Her memories of post-WWII Denmark became video files, stored in the project's hard drives.

Fast forward 1 year. The conversation becomes a meta conversation among the research team. We are combing through the archives of this event, searching for snippets to showcase at two academic conferences: *Data Justice* and *Affects, Interfaces, Events*. Trine's video has been a topic of much interest in our ongoing conversations. She is an engaged citizen, telling a poignant story, which makes her video an affecting piece. But how much should we edit this piece? Because her conversation wanders off point frequently and the interview lasted 3 h, we know we need to cut it in many ways to reshape it for the new exhibition. We also discuss how we might remix the video to highlight only certain points. These are natural decisions any journalist, filmmaker, or artist might make. For us, it raised serious questions from an ethical perspective.

First, what is our justification for remixing or altering someone's memory after they've donated it to us for safekeeping? Second, should we show people's memories in a different context than the one in which they made the original donation? What is our responsibility toward the people we've encountered and the data we've collected? Trine believed her contribution would be saved, archived as part of a larger digital preservation project. She believed her story would remain whole. She believed it would be accessible in the future. Of course she signed a consent form and agreed to future transformations, but to what extent should curators and archivists take responsibility for developing the public's understandings of digital preservation? An ethic of care means more than just meeting needs or expectations, but, as characterized in design disciplines, “doing so in a manner that is attentive, responsive, and respectful to the individuals in need of care.” (see also Edwards and Mauthner, 2002; Engster, 2005; Luka and Millette, 2018). Avram et al. (2019) suggest this both complicates and requires “fundamentally dialogic and adaptive tinkering that defies a factual evaluation or judgement of practice.”

After much debate, we agreed that even with these ethical troubles, we should still show pieces of this video conversation. Remixing Trine's memories into a montage of sound and images, through glitch art techniques, would highlight the illusion of data as an obdurate or secure object. Our goal was to address the myth that massive-scale data collection yields accessible data or usable archives. Trine's case could help us trouble the concept of data itself, the limits of digital preservation, and the precarious future of memory and heritage in a world of continually changing data storage and decoding formats.

Methodologically, the following weeks involved editing the narrative considerably, to find a few minutes in the video that we believed represented the heart of her story. We also played with various statements in Trine's narrative that were completely (or seemingly) unrelated to her memory of the German occupation of northern Denmark, to highlight the challenge of identifying relevance, not for viewers but in terms of the context of the lived experience of events in the 1940s and the later lived experience of recording a memory for future digital preservation.

None of our ideas included showing the video in a straightforward way. Although we had her consent, we considered that showing it in that way could not do justice to her story. We kept this ethical question on the table, iteratively discussing the impact of altering and retelling her story for our own ends—that is, presenting her face and voice to elicit an affective response from people in an entirely different context than her original contribution. Part of this discussion involved flipping the ethics discussion to the other side, whereby we acknowledged the potential positive impact of glitching Trine's memory. After all, our experiment was intended as a critical commentary for the public to see how “accurate” or “complete” data preservation is impossible, for many reasons potentially beyond the control of any single stakeholder.

A few weeks and conversations later, one of the authors contacted Trine and discussed our interest in her story. She was open and interested in the questions and curious about what our next step would be. We met with her two more times and, with her consent, started developing an art installation that would experiment with what algorithms had to say about her memory.

## *Memory Glitch*: Experimenting With Algorithmic Memory-Making

**Figure 1:**
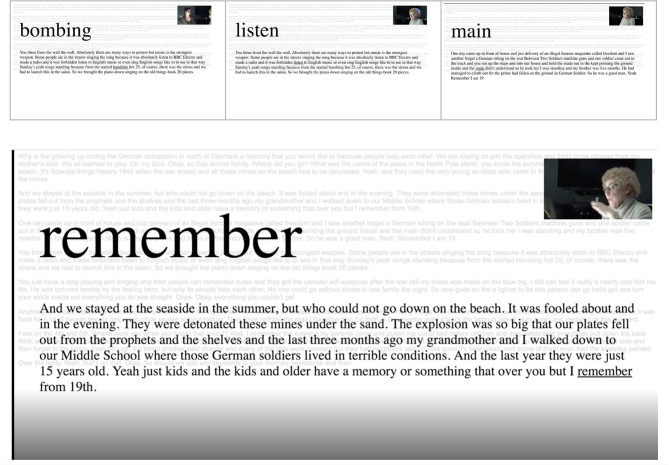
Still shots from video demonstrate output of Google Cloud Speech, v1p1beta1, extended video model. Transcript is aligned with Gentle and displayed with active word and paragraph in sync with video. Fourth image shows closer view.

**Figure 2:**
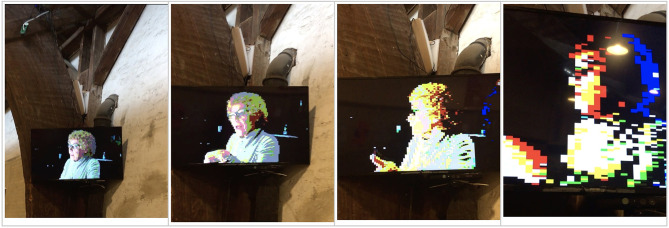
Four progressive snapshot images of the video as the viewer walks closer to the screen, which is suspended from the wall in the exhibit space. A Kinect infrared camera is used to transform the image through pixilation as the viewer moves toward or away from the screen.

**Figure 3:**
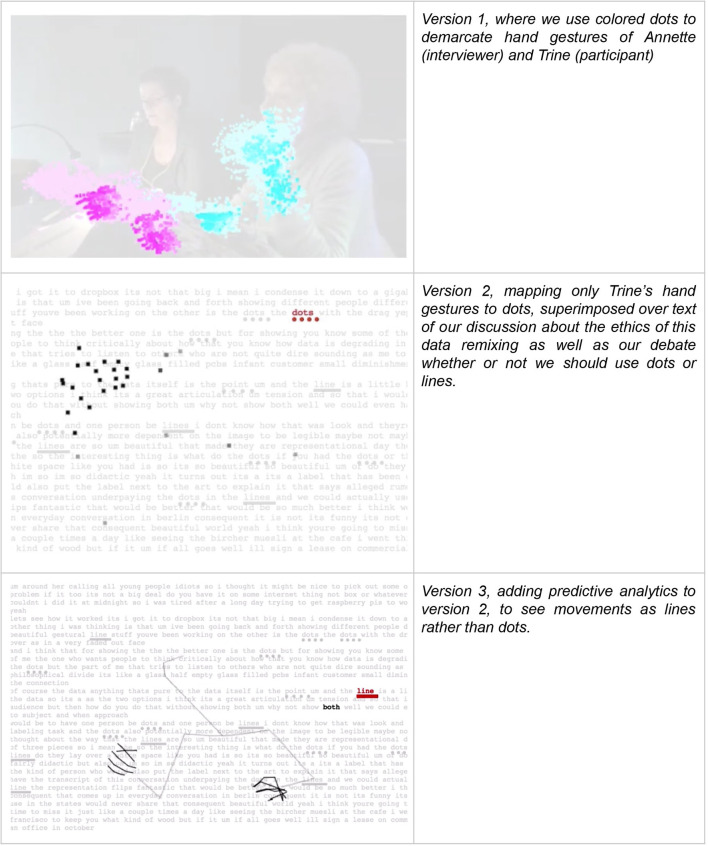
Still images of three variations of code to produce video clip that focuses only on hand movements of participants in the interview. Dots represent presence of a hand in the visual field. Predictive analytics anticipates where the hand will move and produces clusters of dots that lighten in color as other motion is detected, illustrating motion (version 1) or, as the target of the predictive algorithm is refined, discrete lines that approximate fingers (version 3).

The installation, entitled *Memory Glitch*, included three flat-panel displays, which were placed sequentially in the corridor of a public cultural center in Aarhus, as part of the *Affects, Interfaces, Events* conference. The screens present (retell, remix) Trine's story as seen from an algorithmic perspective. We show some still images below from the sequence of screens: *Memory Glitch 1* (Figure 1), *Memory Glitch 2* (Figure 2), and *Memory Glitch 3* (Figure 3). All images are reproduced here with Trine's written and verbal informed consent.

The first screen uses an automated transcription algorithm from Google's machine learning API. Voice is recognized through a series of mathematical operations through a series of deep-learning neural network algorithms. A continuous sampling of sound waves and comparison to thousands of other wave forms produces words, displayed if they meet the defined confidence value. Trine's story thus becomes text, synced to her voice, including all mistranslations and errors.

As viewers listened to her voice and watched the live transcription on the screen, they could begin to see how the text was not a seamless transcription of the audio. It was, at times, difficult to comprehend. The transcribed words were enlarged and flashed on the screen in a sequence that sometimes—but not always—matched her spoken words. Trine's story thus acquired different scales.

*Memory Glitch 2* was an interactive screen. We used a Kinect infrared camera to calculate presence and movements in the corridor around the video. As the viewer moved closer to the video, the pixelation of the image increased. Thus, if viewers wanted to get closer to the video to see the picture or hear the sound more clearly, the fidelity of the visual information would be lost. We conceived this piece to demonstrate the inherently fraught experience of working through any digital archive, where one would encounter the impossibility of truly grasping a full picture. Indeed, as one of the onsite curators noted, as the viewers move closer, the image shifts from a representation of Trine to a representation of the viewers themselves. The camera's infrared sensors, pointed at the viewers, use their body heat to glitch the video.

The final screen in the series, *Memory Glitch 3*, was produced by a Machine Learning algorithm (OpenPose) that detects body keypoints in the image. We used the algorithm to mark the hand movements in the video, thus visualizing gestures. As Trine talks, her hands express, emphasize, and capture an oblique perspective of her narrative. The coding we play with in this piece alternates from dots to lines, which have different visual effects. Both are responses to the predictive algorithm's analysis of where her hands will go.

As viewers watched the screen and listened to the conversation, dots and lines played across the screen, appearing and disappearing in seemingly random ways. These were her hands, moving across the screen. The vocal became gestural. But for most viewers, as we hoped, it was challenging to know what was happening on this screen. Because the algorithm analyzed every frame of the video separately, Trine's gestures were combined at times with her interlocutor. Many viewers asked the researchers, functioning as museum curators at the exhibition, “What is this video supposed to be showing?” For them, the content was obscured. This engenders a double poignancy for us as designers and data scientists who created this rendering. On the one hand, we could feel both the loss of fidelity to the original video story and the emerging beauty of the dancing lines and dots. On the other, we knew that for Trine and possibly others trying to access this as a “clear” representation of her story, it was impenetrable.

Showing three versions of the same video—none of which gives the viewer a logical tale, effectively challenges any simple notion of both the memory and the process of remembering or forgetting. In these videos, the aesthetics, the context, as well as the algorithms transform the original data—already itself an abstraction from the lived experience—into something different. Once memories are put into the world, much like data, they're at risk of being lost, because they have been transformed. To push this reflection into considerations about the datafication process behind the reconfiguring of memory, we offered additional questions in written curator notes alongside the exhibit: What is the relation of the person to the algorithm, vis a vis their personal memory or memory making? What do our personal archives look like when they become data? How do automated processes influence and govern not only what we remember, but what we eventually will see when we try to access a digital memory? What are the ethics of these transformative processes of data science?

## Critical Reflections on the Algorithmic Rendering of Meaning and Memory

This *Memory Glitch* experiment examines how data are not just made but will continually transform throughout their lifespans. The starting point for algorithmic processing is the creation of the data object. As critical algorithm scholars focus on the complications of the algorithmic in machine learning processes, we cannot leave aside or forget the matter of where the data itself originates and how its transformation from lived experience to a computational form is an alteration from the untidiness of everyday life (Cheney-Lippold, 2017) into a measurable unit of cultural information, “flattened and equalized” (Markham, 2013) so it can be made comparable with units of cultural information from other instances and contexts.

How might we learn more about what is being condensed or flattened by reversing this process? How might we conceptualize data as lived experience (as well as within lived experience)? The expression ‘lived experience' has been of particular interest to ethnographers and phenomenologists (cf. van Manen, 1990). Here, we use the term as it has become more colloquially understood, to refer to the whole of sensory and experiential being-in-the-world. In terms of digital and social media use, or the use of platforms to engage in communication and interactions or build/maintain social relations, lived experience also references how this is accomplished with and through digital media on an everyday level, which complicates how we might think about various sensory/physical, affective/emotional, and cognitive processes and modalities. While we don't rehearse the longstanding theoretical discussions around this complication (which has been covered too extensively to even summarize here), we do want to emphasize that anything we might call “memory” is only and always embedded in, created by, and experienced through lived experience.^3^

Our exploration of Trine's video required us to understand how memory was being reduced, simplified into units that would be recombined later, a process we now simply call datafication. We start with the classic idea, made again popular in the edited collection by Gitelman (2013), that data is always already cooked, meaning both that it has been generated according to human values and decisions and also that it only exists because it has been abstracted—or artificially severed from—the context in which it originated. Once objectified, the data is compiled with other units of cultural information, which enables us to do certain things with it, or think certain things because of a larger scale analysis.

Reconnecting the data to the person was an essential step for us to recognize what the original disconnect may have done (or may be doing) to the lived experience that led to the construction of the data form itself. What decontextualization occurs and with what possible consequence?

Looking at Trine's story being transcribed in *Memory Glitch 1*, for example, we start to see—especially through the transcription errors—the importance of her accent, the inflection of her words, among other nuances that Google's transcription services fail to notice. Likewise, in *Memory Glitch 3*, as Trine's hand gestures are highlighted by foregrounding them as data points flowing across the screen, other elements of the situation are blurred. If we focus only on the verbal content of her story, the emphasis and urgency of her telling is erased. The cultural, affective, lived experience of Trine becomes visible through those transformations because they never completely represent what we would expect.

Shifting this point slightly, once we reconnect the data object to the body, story, and person of Trine, we begin to see the flaws in both the data form and the code used to decipher and re-present it. This becomes particularly poignant in *Memory Glitch 2*, where the presence and movements of the viewer directly changes the way the data is decoded in visible form. The observer, archivist, or data archeologist can watch how their body heat functions as an algorithmic layer, overlaying new instructions, effectively obscuring previous instructions, generating a visual that changes as the viewer's body changes. The memory Trine imagines she preserved in digital form morphs again and again into a funhouse mirror image of the body literally viewing it.

In all three video glitches, the boundary we may at one point in time draw to demarcate what counts as the relevant data object might be redrawn entirely differently at some unknown later point in time, when some other aspect of the recording becomes salient. Multiple elements are plausible markers of relevance—words spoken, geotags in the metadata, hand gestures, or the interviewer's critical remarks about the current political party. The missing element will (arguably always) be the meaning in the moment of the retelling.

In these three video renderings, we illustrate only parts of what is presumed to be a whole. And through this partiality, we both recognize and emphasize that a full memory could never be actualized. At such a point, a different formula would be applied to the record to draw a boundary around a different object to call it “data.”

In this analysis, data objects—when severed from their contexts with all the associated affective connections—add (yet) another level of abstraction from the lived experience, even as they represent essential elements of the lived experience. This not only reiterates Boyd and Crawford's (2012) point that “taken out of context, Big Data loses its meaning,” but also goes a step further in identifying how this process takes place, and how it happens when it happens. “Contexting” is the term used by Asdal and Moser (2012) to discuss how humans construct contexts continuously and experimentally, by which certain things are taken as explanatory contexts for others, and these processes are quite variable and political. Certainly this is what we are emphasizing when we foreground the context originating in the datafication and simultaneously remove or relegate to the backstage the multiple contexts preceding this datafication—those involving Trine's lived experience, followed by her donating her story as a memory we should not forget, followed by our repeated viewing and discussion about this video in our research team, and so forth. This analytical move is useful in that it juxtaposes different contexts, as well as different possible futures, confronting the contemporary “taken-for-grantedness” of data, which presents an imaginary of data analysis as impersonal, apolitical, and, because it is—or claims to be—aggregated and anonymized, separated from its origins and effects on human bodies.

At the same time, this exercise helps us see how any human or algorithmic codec will reconstruct a memory based on a particular set of constraints. This is not only a computational but a distinctly human issue, whereby facts are always after the fact, a matter of retrospective sensemaking (cf. Weick, 1969). In this double hermeneutic loop, we recognize how all forms of algorithmic sensemaking involve manipulation of data and transformation of meaning.

One way to specify the calculus used to make decisions at the level of encoding as well as decoding is to separate the algorithm from the algorithmic. An algorithm is generally considered machinic (vs. human) and in computer science traditions is an “abstract, formalized descriptions of a computational procedure” (Dourish, 2016). More broadly, as Cheney-Lippold (2011) notes, algorithms function as inference systems. In the latter conceptualization, what an algorithm does is more important than what it is, a point well-articulated by Gillespie's (2014) idea that algorithms generate or facilitate particular “knowledge logics.” This emphasizes the work algorithms do. As Gillespie adds in 2016, “What makes something algorithmic is that it is produced by or related to an information system committed (both functionally and ideologically) to the computational generation of knowledge or decisions” (p. 25–26). The algorithmic intervenes in terms of step by step procedures. These procedures are formalized and automated. In computational settings, this automation helps the algorithm work “instantly, repetitively, and across many contexts, away from the guiding hand of its implementers” (Gillespie, 2016, p. 26). The process, which involves many stakeholders and systems beyond just the algorithm, builds possibilities for particular futures while simultaneously limiting other options. To return to the point made earlier about the difficulty of identifying agency in this process, Markham et al. (2018) conclude that “We can call this complication of locating moral agency and responsibility a wicked problem. There are no straightforward boundaries, definitions, or answers. Rather, there are only questions to be continually addressed” (p. 6). What our analysis helps us see is that this difficulty stems from our understanding that whatever functions algorithmically is not embedded in a location or element, but in relations (Magalhães, 2018). It is not an object or thing, but a set of process with/in contexts (Seaver, 2015; see also Dourish, 2004).

## Memory, Ethics, and Future-Making

In *Memory Glitch*, we link the algorithmic to the process of making data. These decisions are quite often hidden within the features and affordances of digital services themselves. Apple Memories and Google Photos are powerful tools, helping us store and organize, remember or forget. The problem is that for users, as well as these companies, “remembering” takes center stage, rather than the “forgetting,” what is left out, or what will be omitted in future renderings. In *Memory Glitch*, we used three different predictive data models to classify, in different ways, Trine's experience. As the algorithms used their own pre-made (limited) categories, her experience was flattened (and/or expanded)—retrofitted into the logic outlined by the data models. Rouvroy (2013) would go as far as to say that “the subjective singularities of individuals, their personal psychological motivations or intentions do not matter. What matters is the possibility to link any trivial information or data left behind or voluntarily disclosed by individuals with other data gathered in heterogeneous contexts and establish statistically meaningful correlations” (p. 11–12). Trine's embodied presence and memory is replaced by her “statistical body,” which ultimately functions as “de-territorialized signals, inducing reflex responses in computer systems, rather than as signs carrying meanings and requiring interpretation” (Rouvroy, 2013, p. 4).

Memory does not exist unproblematically (if at all) in the data traces we leave. Of course, even as we say this, we recognize that these traces of data carry the potentialities of remembering. We're not arguing that there is no value in these different renderings of memory, and the different futures they produce. We're suggesting, instead, that memory can't be contained by an artifact because it is always in the relations, in the connections, in the process. And because the memory is always different than the object of the memory.

This is a different approach toward data ethics than the one taken by Metcalf and Crawford (2016), who analyze research practices in data science and make the argument that researchers often “represent themselves as dealing with systems and math, not people—human data is treated as a substrate for testing systems, not the object of interest in itself.” (p. 3) Metcalf and Crawford help researchers think about the origins of data by positing “data are people,” which may help protect the persons who (often unwittingly) participate in big data experiments. Our questions turn in a different direction. Through the video installation we are trying to direct attention to a different level of impact, whereby we're not as focused on the typical ethics question of whether or not we are harming people through various forms of data collection or analysis, but rather on: *what possible futures are being enabled or disabled*?

We're also not asking what ethical or moral principle is being used in different moments or by various stakeholders in the data science processes of data archiving and digital preservation, but rather: What sort of ethic is being *produced*? Markham (2015) reminds us that any creation of a data object constitutes a choice about what counts as data and what is discarded as non-relevant. In this action, we're building the ethics of the future. When the creation of a data object generates or attends to only certain elements of experience, to what extent has this already manipulated lived experience? Or are we simply manipulating the representation of lived experience: its memory, future, etc?

In Trine's case, she wanted her experiences of WWII to be remembered so these memories could create a better world, where people remember the atrocities of the war and respect and help each other. But once this memory is datafied, her desire about what this data means, or how it should be interpreted by future viewers/listeners/readers, is separated from the objects that are retained. Once the decoder ring—the sensemaking logic—is detached, meaning becomes a floating signifier, up for grabs. To draw on Theresa Senft's (2008, p. 46) apt turn of phrase, the notion of “the grab” is evocative because it emphasizes how anything we take to be real— in a world of digital/data objects and endless copy/paste possibilities–is the outcome, not of gazing, but grabbing. As she says:

To grab means to grasp, to seize for a moment, to capture (an object, attention), and perhaps most significant: to leave open for interpretation, as in the saying “up for grabs.” What is grabbed, like a screenshot, is just that, a moment frozen in time for inspection. The material, affective, embodied, lived part of this is never singular or just a 3D version of the screenshot. What is seen indicates what is not seen. Accidental or intentional, the grab still has impact. And has an ethic (Senft, 2018).

In *Memory Glitch 1, 2, 3*, a confluence of entities, processes, and decisions create a momentary stillness. To be sure, the case of Trine's memory being transformed or reconfigured is common. It depicts the almost by now banal disconnect between what people expect their digital archive to be and what actually is available and rendered over time. Yet when the exact same dataset is presented in multiple transmogrified forms that each tell a different story, this set of videos creates a moment for reflection. Viewers and developers alike can consider the potential violence (Hoffmann, 2016) of automated machinic processes on people whose memories are impacted. On the flipside, they can also imagine their role as an interactant with the algorithm as an active, if mysterious partner, which Magalhães (2018) contends can lead to greater, not less ethical agency for everyday users.

This is a matter of impact. And a question about what kind of analysis and models do we want to produce, to generate a better set of future ethics? The models we construct through data analytics cannot be separated from the futures they build. Focusing critical ethical attention on future practices and technologies that may render historical meaning in unexpected ways can help data scientists, consumers, and companies understand the impossibility of mapping data to memory in a one to one fashion and identify various algorithmic agents in the process of digital memory making. Creative and artistic play with algorithmic possibilities, for everyday users, can build more nuanced considerations of what a future holds when we have interpersonal, intimate relationships with autonomous nonhuman entities that function on our behalf. What do these relations entail? And if, after understanding the impossibility of preserving memory as data, we still want to preserve memories in ways that give us a sense greater fidelity to the original lived experience, what sort of “digital decoder rings” should be included to help future viewers (try to and likely fail to) understand our contexts?

A critical data science, we argue, can use its strengths at building creative algorithmic processes to create interventions like ours that help reveal the potentiality for generating new meaning as memories are manipulated through automated systems. This can have both enabling and constraining potentiality.

## Author Contributions

Both authors listed have made a substantial, direct and intellectual contribution to the work, and approved it for publication.

### Conflict of Interest

The authors declare that the research was conducted in the absence of any commercial or financial relationships that could be construed as a potential conflict of interest.

## Footnotes

^1^For more details, see Rehder and Ostrowski (2017), Bratton et al. (2016); Markham (2019), or the project website (*https://futuremaking.space/morm/*).

^2^This stance, as articulated by Luka and Millette (2018), emphasizes “the integration of feminist and intersectional values into considerations of data analyses, including big data” (p. 4). In critical data studies or critical data science, if we follow the work of Hoffmann (2016, 2018), this means not only centralizing ethics but also considering how data construction, data modeling, and data processing might conduct violence on people, symbolically, culturally, or physically.

^3^At the same time, *Memory Glitch 1, 2, 3* compels us to flip this idea to consider that anything we call data is also memory. Literally, when data occupies space in a computer, it is called memory. But the computational concept of memory is that it is useful information for performing, and more importantly, recalling and repeating certain operations.
